# Perceptions of Dental Medicine Students on Equity within Healthcare Systems in Romania: A Pilot Study

**DOI:** 10.3390/healthcare10050857

**Published:** 2022-05-06

**Authors:** Sorin Hostiuc, Oana-Maria Isailă, George-Cristian Curcă

**Affiliations:** 1 Department of Legal Medicine and Bioethics, Faculty of Dental Medicine, “Carol Davila” University of Medicine and Pharmacy, 020021 Bucharest, Romania; sorin.hostiuc@umfcd.ro; 2 “Mina Minovici” National Institute of Legal Medicine, 042122 Bucharest, Romania; cgcurca@yahoo.com; 3 Department of Legal Medicine and Bioethics, Faculty of Medicine, “Carol Davila” University of Medicine and Pharmacy, 020021 Bucharest, Romania

**Keywords:** equity, resources, disadvantaged population, future dentists, healthcare

## Abstract

The scope of this paper is to evaluate the opinion of future dentists on equity within healthcare systems from a social and medical perspective. Material and methods: We conducted an observational study based on a survey among year five students from the “Carol Davila” Faculty of Dental Medicine Bucharest using an online questionnaire composed of graded answers to 14 statements on the theme of equity within healthcare systems before taking this course. Results: The questionnaire was sent to 300 students, of whom 151 (50.3%) responded; 79.47% of these were female and 20.53% were male; 9.3% had a rural background and 90.7% had an urban background. The majority of respondents expressed strong agreement that equity in public healthcare and acknowledging disadvantaged populations was important. The majority of students also strongly agreed that inequity came about from a lack of accessibility to medical care, lack of financial resources, and the absence of a second medical opinion. There were no statistically significant differences specific to the gender and background environment of the respondents. Conclusions: The notion of equity is known to future dentists. However, contextual clarifications of the concept itself and its adequate quantification are necessary.

## 1. Introduction

The right to healthcare is a fundamental human right [1]. To ensure this right, medical resources are required as well as acknowledgment of the different needs of population groups and individuals. Despite the measures taken and the awareness of this problem, equity within healthcare systems still seems to be a utopian desideratum, complex and difficult to manage, conditioned by a multitude of factors such as geographic, economic, social, cultural, and biological [2]. The concept of primary health care (PHC), according to WHO, “addresses the majority of a person’s health needs throughout their lifetime”. This includes physical, mental, and social well-being and is people-centered rather than disease-centered. It is a whole-of-society approach that includes health promotion, disease prevention, treatment, rehabilitation, and palliative care. A primary health care approach includes three components: meeting people’s health needs throughout their lives; addressing the broader determinants of health through multisectoral policy and action; and empowering individuals, families, and communities to take charge of their own health. By providing care in the community as well as care through the community, PHC addresses not only individual and family health needs, but also the broader issue of public health and the needs of defined populations.” [3].

The right to healthcare can sometimes be conditioned by equity. Equity is a difficult concept to evaluate directly. It is defined by WHO as ”the absence of unfair, avoidable or remediable differences among groups of people, whether those groups are defined socially, economically, demographically, or geographically or by other dimensions of inequality (e.g., sex, gender, ethnicity, disability, or sexual orientation).” [4]. It is correlated with fairness and justice, norms that are present in every society but which vary among different cultural values [5,6]. Equity can have theoretical and practical significance, which differ for every person and, as opposed to equality, equity is normative and based on value [7]. In healthcare, not everything that is unequal is also inequitable (for example, the increased presence of breast cancer in women; young people having better health than old people); and not everything equal is implicitly equitable when compared with a person’s individual needs [6]. Equality is easily quantifiable because it targets distributed resources and not necessarily the real needs of each person from a biological/psychological/social standpoint.

Factors that produce inequity within healthcare systems can be: geographic location, the privilege of certain population groups to the detriment of others, race/ethnicity, gender, sexual orientation, education level, income, profession [8], some interpersonal contexts of a familial, social or professional nature which can place a person in a disadvantaged or inferior condition, undermining his or her autonomy and capacity to resort to the medical services they require, for example in the case of domestic violence victims [9].

The dental healthcare system on a national level in Romania is predominantly private. The latest statistics show that among dentists, 2771 worked in the public sector and 15,720 in the private sector. Also according to these statistics, the number of independent medical dental offices was six times smaller in rural areas than in urban areas. Theoretically, in rural areas there were 6.4 times more people per dentist than in urban areas. In some counties, the number of dentists registered in the public system was less than ten [10], and most counties have between 300,000 and 499,999 inhabitants [11].

The principle of equity in healthcare refers to distributive justice through the lens of social correctness. It targets, in general, persons who are already unfavored in the lottery of life, people who, in addition to the need for medical care, are subjected to further disadvantage [6].

Resource allocation in healthcare systems, defined as the distribution of goods and services, can be on a geographical level, on a health care unit level, on a physician level and/or on a patient level [12,13].

This paper aims to evaluate the degree of acknowledgment and sensitivity of future dentists and the need for informative and educational programs relating to equity within healthcare systems from a medical and social perspective, referring to primary healthcare aspects in Romania in scenarios from the regional level to patient level according to the relevant resources prioritization.

## 2. Materials and Methods

We performed an observational study based on a survey on year five dentistry students from the ” Carol Davila” University of Medicine and Pharmacy Bucharest. We developed a questionnaire of 14 statements with 5 Likert-type graded answers which presented different scenarios on inequity in healthcare systems. 

The study received the institutional ethics committee’s approval code 972/26 January 2021. The validated questionnaire was uploaded on the e-learning platform, and was available to respondents before they attended the course on resources allocation in healthcare systems. Answering the questionnaire was optional. We used Microsoft Excel 2007 to collect the data and Jamovi 2.2.5 to statistically analyze it. To analyze the categorical variables, we used the Chi^2^ test, and a value of *p* < 0.05 was considered statistically significant.

## 3. Results

### 3.1. Demographic Characteristics of the Respondents

The questionnaire was available to 300 students, of whom 151 (50.3%) responded. Females accounted for 79.47% of these and males 20.53%. In respect of origins, 9.3% had a rural background and 90.7% had an urban background. The average age of the respondents was 23.5 years old.

### 3.2. Opinions on Equity

The first three statements concerned disadvantaged populations and equity from a general point of view. The fourth statement targeted the conceptual difference between equity and equality in the opinion of the respondents. The respondents’ opinions were as follows (Table 1):

Statements 5 to 14 depicted scenarios to evaluate the sensitivity and conceptualization of the notion of equity depending on the patients’ social and cultural background.

Statement 5:“It is inequitable if a person from a rural area needs dental treatment but in the respective village there is no dentist and the patient does not have the means to travel to the nearest city or town for treatment.” 6 (4.0%) strongly disagreed, 7 (4.6%) slightly disagreed, 13 (8.6%) had a neutral opinion, 33 (21.9%) slightly agreed, and 92 (60.9%) strongly agreed.

**Figure 1:**
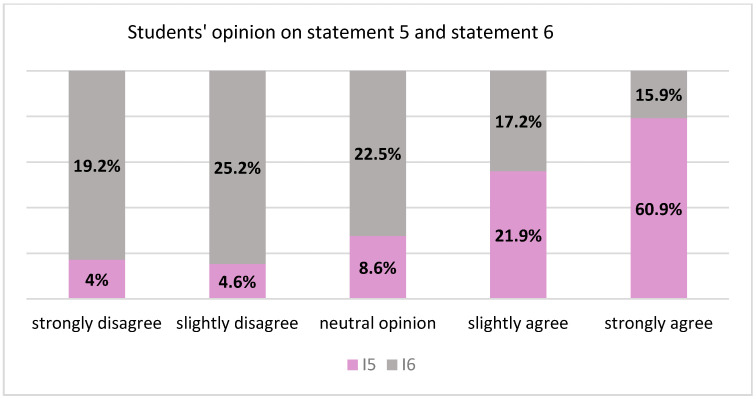
Opinion on statement 5 vs. statement 6.

Statement 6: “It is inequitable if a person from a rural area needs dental treatment but in the respective village there is no dentist and the patient has the means to travel to the nearest city or town for treatment” 29 (19.2%) strongly disagreed, 38 (25.2%) slightly disagreed, 34 (22.5%) had a neutral opinion, 26 (17.2%) slightly agreed and 24 (15.9%) strongly agreed (Figure 1)

**Figure 2:**
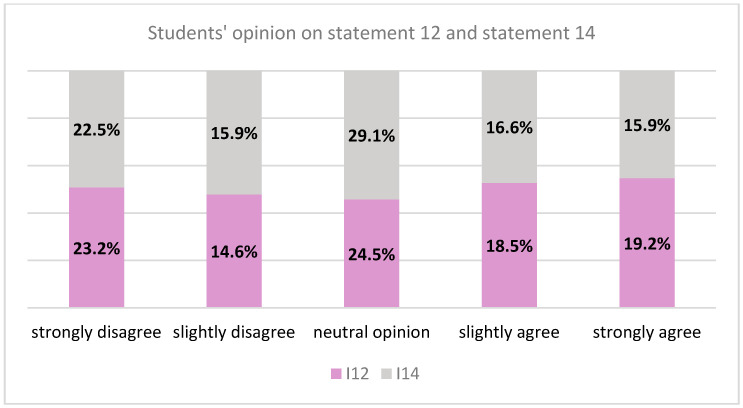
Opinion on statement 12 and statement 14.

For statements 7–11 and 13 the answers were as follows (Table 2):

Statement 12: “It is inequitable if a victim of domestic violence refuses to ask for dental treatment any more because the dentists always accuse them of not respecting medical indications and dental appointments.” Question 14 states: “It is inequitable if a dentist prioritizes treatment for a woman abused by her husband.” This refers to victims of domestic violence, a vulnerable population group. The respondents’ opinions were the following (Figure 2):

On all the aforementioned statements there were no statistically significant differences in respect of the gender and environmental backgrounds of the respondents.

## 4. Discussion

This study exposes the dental students’ opinions on equity in the healthcare system, before they attended the course on this topic.

The answers to the first three statements, which targeted the elementary perception of the existence of disadvantaged population groups and the concept of equity, indicated an awareness of these aspects. The majority of future dentists agreed to the existence of disadvantaged population groups; that their health status is poor; and that equity is important in healthcare systems. The study conducted by Wilson et al. had similar results among medical students [14]. Fairbrother et al. in focus groups on young people from disadvantaged regions in England found that they perceived the behavioral impact of health disparities and their social determinants [15]. 

Statement 4 targeted the conceptual delimitation of equity versus equality. In the present study, it was discovered that there is a tendency in the majority (42.4%) to equivalate equality with equity in the presented scenario. Equity is difficult to quantify and standardize on a personal level because of the different needs of each individual from a medical as well as a contextual point of view; thus, the conceptual confusion of equity versus equality is present [7]. In an attempt to depict quantifiable equity, in the past, there have been various definitions including the equality of expenses per capita, the equality of offer per capita, the equality of offer for equality of needs, the equality of accessibility for equal needs, the equality of use for equal needs, the equality of marginally satisfied needs, and the equality of health [16]. Thus, it has been observed that, situationally, equality can be applied in the definition of equity without the two being equivalent since the ratio of needs/offer has priority. On the other hand, equity can lead the path to social equality in some contexts [6]. Equal accessibility to medical services may equate to equal opportunities but this would only be a step towards equity, being also necessary to have equal opportunity in requesting medical services and using them, elements which cannot be equalized [16].

Statements 5 and 6 were similar, the difference being the financial level of the person who needs medical care. In the situation of the disadvantaged person from the rural environment with reduced accessibility to dental care and lack of financial resources, the majority of the respondents (60.9%) expressed strong agreement that this was inequitable. For the same situation, with the changed variable of the presence of financial resources, the majority of the respondents (25.2%) expressed disagreement, considering that this is not inequitable, followed by 22.5% who were neutral. Thus, the importance of patient individual allocation of resources (individual financial status) was highlighted among the respondents, undermining a scarce regional allocation. Romania is among the countries with inequitable access to health care, which leads to hospital costs for pathologies that could be prevented or treated on time. The majority of the population groups affected in this sense in terms of geographical, social, and/or financial impediments are the rural, ethnic groups, with a socioeconomic level below subsistence [17]. When demand surpasses supply, the allocation is problematic; solving the disparities depends on the nature of the scarce resources [18]. From the standpoint of an allocation model based on social services, the medical professional’s duty to offer care is limited by the resources that the state provides [19]. In the depicted scenario, location can also be a resource. The model based on social services focuses on the principle according to which a right has as a result, real or potential, the well-being of the majority [12,18], a utilitarian approach with assumptions that are not always equitable on an individual level [13,18].

Studies that have analyzed equity in the healthcare system, also from a locative perspective through the lens of the regional allocation of resources, have focused on the rural-urban differences and the distance from health units, and have found that the accessible location of the medical unit has an important role in ensuring equity [20]. 

Also related to the aforementioned scenarios, income inequalities are an issue that increases inequity for economically disadvantaged populations in healthcare systems [21].

Richard et al., in a study which analyzed the equity of access to primary healthcare for vulnerable populations, found that most efforts to improve this access were aimed at facilitating the supply-side determinants without focusing on also improving the demand-side determinants, which need a complex approach [22]. 

For statements 7, 8, 10, and 13, the majority of the respondents have acknowledged the inequity based on income level, lack of employment, ethnicity, personal needs, factors that are well known in the specialized literature on this topic [4,5]. A healthcare system based on the Rawlsian approach of “equal opportunity” creates the premise of equity for all members of society [23,24]. Individual, inalienable factors such as the ethnicity of a person requiring medical treatment can be determinants of inequity expressed by discrimination, elements which cause a loss of trust and addressability of the patient, perpetuating and increasing inequity, [25]. a phenomenon which, according to some authors, is “institutional racism” [25,26,27]. 

The need for a second medical opinion, according to the study of Shmueli et al., derived from the patients’ uncertainties about diagnosis and treatment on the background of unclear communication in the physician-patient relationship. Cost, accessibility, clinical and behavioral issues in seeking a second medical opinion are elements that have an impact in equity in healthcare systems [28].

For statements 9 and 11, there was a majority of neutral opinions. These referred to scenarios in which a professional group benefitted from medical services without mentioning if this aspect was to the detriment of other population groups (statement 9)such as that of a dentist reducing the cost of medical treatment to attract patients, without giving details of other contextual elements in the sentence of the scenario (statement 11). The answers provided indicate the marked sensitivity of the respondents to situations that clearly show the disadvantage of the patient/potential patient, and a lack of sensibilization regarding a potential inequity on favorable grounds in this regard.

Statements 12 and 14 exposed scenarios in which the patient was a victim of domestic violence. The majority of respondents had a neutral opinion but the percentages for each answer options were similar. The victims of domestic violence bear the consequences of gender inequality [29] and require a particular approach within healthcare systems and implicitly within the physician-patient relationship. They can be non-compliant patients because their abusive partners can hinder them from following medical indications or respecting medical appointments [30]. This is contextual, forced noncompliance. The interference of the aggressor within the physician-patient relationship is a form of control that affects the autonomy of the victim and their rights to medical care. Thus, the important constituents of the physician-patient relationship are represented by acknowledging the phenomenon of domestic violence; providing necessary medical and social assistance, which is urgent for the victim; and respecting the victims’ autonomy, which is undermined in the domestic environment [31]. An important role in the equity for patients who are victims of domestic violence is played by the allocation of resources made by each physician, on patients, according to the available resources (including time) and the individual needs of the victim patient [13,29]. The medical examination of the patient must take place whenever it is possible for them. The physician must prioritize vulnerable patients each time it is necessary to do so and provide them with adequate medical care [9,31].

The limitation of the present study is represented by the small and not very differentiated sample. The study results can be useful to teachers who have healthcare staff in their educational care. Approaching the aspects of equity focused also on how the patient’s background, needs and resources can alleviate the disparities in healthcare systems by making the physician aware of any “unspoken parts of the story” of the patient. At the same time, empathetically covering this personal information gap would increase people’s confidence and compliance with healthcare systems.

## 5. Conclusions

This study reveals the need for constant informative programs for medical staff concerning the equitable allocation of resources within healthcare systems, with an emphasis on the allocation of resources by the physician according to the individual needs of the patients and the allocation of resources by the patient in particular contexts, depending on priorities.

The concept of equity is not unfamiliar to future dentists. They are sensitive and receptive to aspects concerning inequity and inequality, which they manage adequately in theory in most cases, but additional contextual clarifications focused on the socio-economic particularities of the patient, are needed in this sense.

## Figures and Tables

**Table 1:** Opinion on the first four statements.

Statement	Strongly Disagree	Slightly Disagree	Neutral Opinion	Slightly Agree	Strongly Agree
S1“The presence of disadvantaged populations is acknowledged on a social level.”	3 (2%)	13 (8.6%)	13 (8.6%)	43 (28.5%)	79 (52.3%)
S2”Equity represents an essential element in public healthcare.”	2 (1.3%)	1 (0.7%)	14 (9.3%)	27 (17.9%)	107 (70.9%)
S3 ”Disadvantaged populations have a poor state of health.”	1 (0.7%)	4 (2.6%)	9 (6%)	41 (27.2%)	96 (63.6%)
S4 “It is equitable for dental healthcare to be free of charge as long as any person would have the right to a single dental appointment every two months.”	7 (4.6%)	13 (8.6%)	37 (24.5%)	30 (19.9%)	64 (42.4%)

**Table 2:** Opinion on statements 7–11 and 13.

Statement	Strongly Disagree	Slightly Disagree	Neutral Opinion	Slightly Agree	Strongly Agree
S7 “It is inequitable if a person who is unemployed cannot benefit from dental treatments they need because they do not have the financial means.”	11 (7.3%)	23 (15.3.%)	37 (24.7%)	34 (22.7%)	45 (30%)
S8 “It is inequitable if a person who is employed cannot benefit from the dental treatment they need because they do not have enough financial means.”	6 (4.0%)	13 (8.6%)	24 (15.9%)	47 (31.1%)	61 (40.4%)
S9 “It is inequitable if the employees of a bank benefit from dental care with reduced costs, promptly and regularly through their place of work.”	17 (11.3%)	27 (17.9%)	39 (25.8%)	37 (24.5%)	31 (20.5%)
S10 “It is inequitable if a person of a certain ethnicity refuses to ask for dental treatment they need because the dentists always approach them with contempt.”	20 (13.2%)	23 (15.2%)	13 (8.6%)	22 (14.6%)	73 (48.3%)
S11 “It is inequitable if a dentist reduces treatment costs to attract patients.”	42 (27.8%)	39 (25.8%)	47 (31.1%)	14 (9.3%)	9 (6.0%)
S13 “It is inequitable if a person is refused a second medical opinion.”	6 (4.0%)	3 (2.0%)	10 (6.6%)	10 (6.6%)	122 (80.8%)

## Data Availability

Data supporting these results can be available online on elearnmed.ro only following an explicit request in this regard.
